# Two Gingival Cell Lines Response to Different Dental Implant Abutment Materials: An In Vitro Study

**DOI:** 10.3390/dj10100192

**Published:** 2022-10-17

**Authors:** Muataz A. Osman, Evgeny Kushnerev, Rasha A. Alamoush, Kevin. G. Seymour, Julian M. Yates

**Affiliations:** 1 Division of Dentistry, School of Medical Sciences, Coupland 3 Building, University of Manchester, Oxford Road, Manchester M13 9PL, UK; 2 Periodontology Department, Faculty of Dentistry, The University of Benghazi, Benghazi, Libya; 3 Restorative Department, Faculty of Dentistry, Libyan International Medical University, Benghazi, Libya; 4 Blond McIndoe Laboratories, Division of Cell Matrix Biology & Regenerative Medicine, Faculty of Biology, Medicine & Health, The University of Manchester, 3.106 Stopford Building, Oxford Road, Manchester M13 9PT, UK; 5 Prosthodontic Department, School of Dentistry, University of Jordan, Amman 11942, Jordan

**Keywords:** implant abutment materials, human gingival fibroblasts, human gingival keratinocytes, cell proliferation, cytotoxicity

## Abstract

***Objectives***: This study aimed to investigate the response of human gingival fibroblasts (HGFB) and human gingival keratinocytes (HGKC) towards different dental implant abutment materials. ***Methods***: Five materials were investigated: (1) titanium (Ti), (2) titanium nitride (TiN), (3) cobalt-chromium (CoCr), (4) zirconia (ZrO_2_), and (5) modified polyether ether ketone (m-PEEK). Both cell lines were cultured, expanded, and seeded in accordance with the protocol of their supplier. Cell proliferation and cytotoxicity were evaluated at days 1, 3, 5, and 10 using colourimetric viability and cytotoxicity assays. Data were analysed via two-way ANOVA, one-way ANOVA, and Tukey’s post hoc test (*p* < 0.05 for all tests). ***Results***: There was a statistically significant difference in cell proliferation of HGKC and HGFB cells in contact with different abutment materials at different time points, with no significant interaction between different materials. There was a significant effect on cell proliferation and cytotoxicity with different exposure times (*p* < 0.0001) for each material. Cell proliferation rates were comparable for both cell lines at the beginning of the study, however, HGFB showed higher proliferation rates for all materials at day 10 with better proliferation activities with ZrO and m-PEEK (40.27%) and (48.38%) respectively. HGKC showed significant interactions (*p* < 0.0001) in cytotoxicity between different materials. ***Conclusion***: The present in vitro assessment investigated the biocompatibility of different abutment materials with soft tissue cells (HGFB and HGKC). The findings suggest that m-PEEK and TiN are biologically compatible materials with human cells that represent the soft tissue and can be considered as alternative implant abutment materials to Ti and ZrO_2_, especially when the aesthetic is of concern.

## 1. Introduction

Over the last 60 years, the field of implant dentistry has markedly evolved to provide long-term successful and predictable treatment outcomes with many biological and mechanical advantages over conventional prostheses [1]. It has also shifted from the surgical placement of implants according to bone availability to prosthetically driven implant planning and placement. This shift has influenced the range of available dental implant materials available to restore single crowns, in partially and fully edentulous jaws [2].

Dental implant therapy requires transmucosal suprastructures to act as a foundation for the restorations [3]. Ti and metal alloys have been, for many decades, used as transmucosal abutments, and the appearance of the grey metallic colour of the implant or its abutment through soft tissue have raised an aesthetic challenge, particularly in the maxillary anterior segment [3,4]. The aesthetic improvement and colour modification of the abutment can be achieved by different means including modifying the Ti material and/or surface or the use of other materials [5].

Recently, ZrO_2_ has been widely used, particularly in the anterior region of the oral cavity [6,7]. ZrO_2_-based implants and abutments are deemed to be biologically inert, with no adverse and/or unfavourable reaction with host tissues after implant placement and abutment insertion [8]. The more aesthetically pleasing colour and reduced bacterial plaque adhesion and accumulation have given ZrO_2_ a wider range of applications in restorative and prosthetic dentistry [9]. ZrO_2_ abutments demonstrate a lesser effect on optical outcomes of peri-implant mucosal tissue, when compared with Ti abutments [10].

CoCr has been historically one of the most used alloys in dentistry owing to its high strength, durability, biocompatibility, corrosion resistance, and bond strength to ceramics. Yet, one of the biggest disadvantages of this material is the effect of the cumulative distortion, porosity, and high labour and manufacturing costs related to the casting processes and structural hardness, which complicates the finishing process of the restoration [2].

Due to Ti’s low strength and its ability to undergo physical abrasion when exposed to the oral environment [11,12], as well as the grey colour that raises aesthetic concerns when it is not adequately masked by soft tissue at the gingival area [13], TiN-coating has been recently introduced to overcome these challenges. TiN-coated dental implants show higher physicomechanical properties [14,15] and allow for better camouflage under the gingival tissue than the conventional grey Ti implants.

Numerous medical devices including dental implants, abutments, healing caps, orthodontic braces, and most notably, and denture prosthetic frameworks have been developed via academic research and commercialization of biocompatible polymers and polymer-based composite materials [16]. Consequently, polyether ether ketone (PEEK) and similar composites have been improved to have a wide variety of physical, mechanical, and surface characteristics that may be tailored to meet the needs of various oral implant applications [17].

At present, there have been some studies on PEEK as a restorative material for fixed bridges, orthodontic brackets, and implant abutments [18,19]. However, when used as an implant body, PEEK has insufficient biological activity and cannot form good osseointegration with the surrounding bone. These defects severely limit the practical and clinical application of PEEK in dental implantology. Therefore, different modification methods that can enhance PEEK’s biological activities have recently become of interest [20].

Early research on PEEK has been controversial and inconclusive in terms of the material’s biological behaviour and biocompatibility. Consequently, different strategies have been introduced to improve the biologic properties of PEEK implants/implant components [21,22,23,24].

A central issue in the long-term success of dental implant therapy is not only related to the integrity of osseointegration around the implant but also influenced by the health of the epithelium and the quality of the connective tissue attachment to the implant abutment [25,26].

HGFBs and HGKCs are the main cell populations in peri-implant soft tissue, and they are crucial for both wound healing and regeneration processes. The number, the biological integrity, and the bioactivity of these cells at the implant–gingival interface may impact the formation and the quality of peri-implant soft-tissue seal [27]. It has been hypothesized that the surface characteristics and the type of materials used in manufacturing implant abutments may be important for determining the quality of the soft tissue seal around the implant abutment [28].

Several in vitro studies with different cell types have been used to analyse cell response towards various abutment materials and surfaces [29,30,31]. However, there are insufficient data on HGKC behaviour and their response to different abutment materials.

Using an in vitro model, this experiment examined how HGFB and HGKC cells’ proliferation and cytotoxicity were affected by different abutment materials with the same surface roughness that represents the currently used abutment materials.

## 2. Materials and Methods

### 2.1. Discs’ Preparation

**Figure 1:**
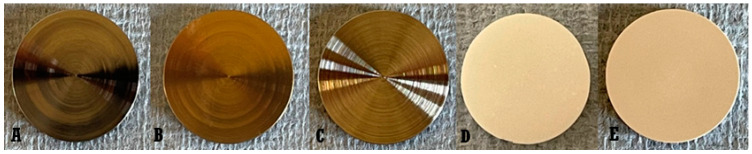
Abutment materials discs (**A**): Ti, (**B**): TiN, (**C**): CoCr, (**D**): ZrO_2_, (**E**): m-PEEK).

Five different materials were selected: Ti, TiN, CoCr, ZrO_2_, and m-PEEK. Twenty-two discs of each material were used in this experimental work (Figure 1). All discs were produced and received from Zimmer Biomet 3i (Palm Beach Gardens, FL, USA). All discs were 14 mm in diameter and 1 mm in thickness.

Five different materials were selected: Ti, TiN, CoCr, ZrO_2_, and m-PEEK. Twenty-two discs of each material were used in this experimental work (Figure 1). All discs were produced and received from Zimmer Biomet 3i (Palm Beach Gardens, FL, USA). All discs were 14 mm in diameter and 1 mm in thickness.

All discs were prepared, cleaned, sterilized, and packaged by Zimmer Biomet 3i (Palm Beach Gardens, FL, USA).

The Ti and CoCr discs were machined on a Star SV-32 Swiss style lathe. TiN coating was conducted via a standard physical vapour deposition (PVD) method, in accordance with the specifications of the manufacturer. The ZrO_2_ discs were machined with an Zfx inhouse 5 axis milling machine using CAM software (Hyperdent V8.2.3) at 14 min milling time per part. The cutting tools used for milling were T20 (92 mm), T15 (1.5 mm), and T10 (1 mm). Furthermore, the PEEK material used in this study was the modified version, where the manufacturer used carbon and glass particles within the PEEK to improve other properties.

All discs were placed in containers, immersed in heavy duty cleaning detergent (Sonicor123, West Babylon, New York, NY, USA), and ultrasonically cleaned for 10 min at 65 °C. They were then rinsed under running tap water until all the detergent was removed. Immediately after, they were ultrasonically cleaned in deionized water for 10 min as a final rinse. Finally, they were dried in an oven for 30 min at 100–105 °C, except for the m-PEEK discs, where the oven temperature was lowered to 60–70 °C, as their heat resistance is lower than the other materials.

Dried samples were then Gamma-sterilized at Gamma dose range of 25–38 kGy and validated to achieve the Sterility Assurance Level of 10-6, except for the ZrO_2_ discs, which were sterilized by autoclaving.

**Figure 2:**
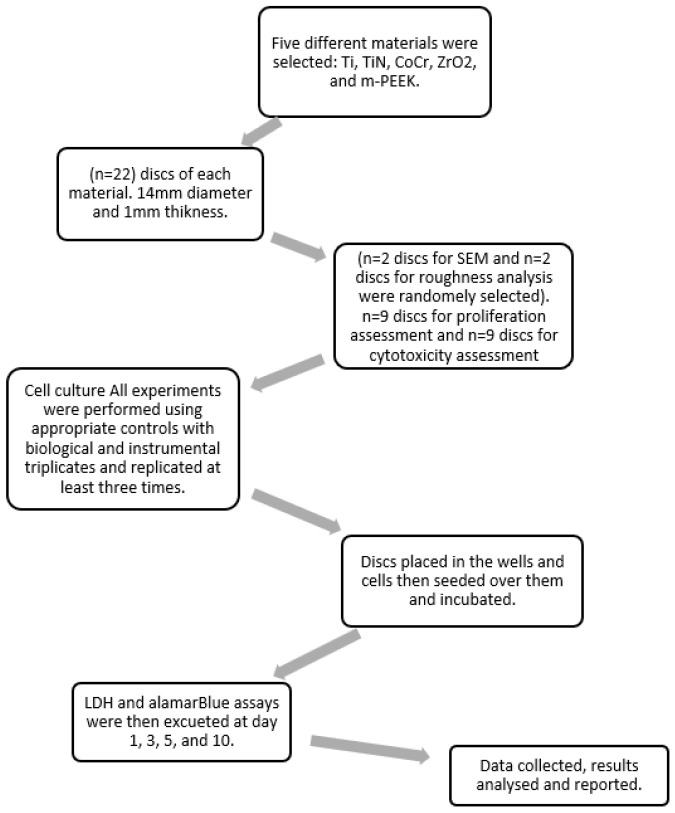
A flowchart illustrating the study steps.

Discs were allocated to different analysis as follows: 2 discs of each material used for surface roughness measurements, 2 discs of each material were used for SEM analysis, 9 discs of each material were used for cell proliferation analysis, and 9 discs of each material were used for cell cytotoxicity analysis (Figure 2).

### 2.2. Surface Roughness Measurements

Two discs of each group were randomly selected and used for surface roughness measurement. Three points on each side of the disc were measured using *Sa* value, and the average was used.

The surface roughness of all discs was measured using a 3D, non-contact, high resolution contour GT-K optical surface profiler (Veeco ContourGT, Tuscon, AZ, USA). The Contour GT-K uses white light interferometry to determine surface topography from nanometre-scale roughness, through millimetre-scale steps with sub-nanometre resolution. Its measurement accuracy is in the single-digit nanometre range and can measure changes in surface height within a single field of view up to 10 millimetres in difference.

To perform the measurement, a vertical scanning interferometry (VSI) mode was selected. A 50× objective lens that provides an area of 174.7 (x) and 132 (y) microns was used. A Gaussian regression filter with a short wavelength cut-off of 25 μm (0.025 mm) was applied before determining the surface roughness parameters. Mean surface roughness (*Sa*) values were used to represent the surface roughness as recommended by the dental implant research methodology and the scientific literature [32,33].

### 2.3. SEM Surface Morphology Analysis

The surface of (*n* = 2) discs of each group (randomly selected) were examined using a scanning electron microscope (SEM) (Quanta FEG 250 SEM, Edificio I + D—Campus Río Ebro C/Mariano Esquillor s/n 50018 Zaragoza—Spain) (Figure 2). The images were obtained using the following parameters: 485 X magnification, accelerating voltage of 20 kV, spot size of 3.0, and working distance (WD) of 7.6–7.9 mm.

### 2.4. HGFB and HGKC Cell Culture Preparation

Commercially available normal primary cell lines were purchased and cultured, in accordance with the recommendations of the manufacturer (ATCC and LifeLine Cell Technology), for HGFB and HGKC, respectively. Standard protocols for cell culture, maintenance, washing, freezing, and thawing were maintained throughout the whole experiment. Both cell lines were grown in their relevant growth media with all the necessary supplements and growth factors, as detailed below.

HGFB were cultured with fibroblast basal medium supplemented with fibroblast growth kit. The final concentration of each component was as follows (HGF complete growth media): L-glutamine: 7.5 mM; rh FGF-β: 5 ng/mL; rh insulin: 5 µg/mL; hydrocortisone: 1 µg/mL; ascorbic acid: 50 µg/mL; foetal bovine serum: 2%.

HGKC were grown in their relevant growth media: DermaLife K Serum-Free Keratinocyte Culture Medium and DermaLife K LifeFactor Kit. The final concentration of each component in complete keratinocyte growth medium was as follows: rh Insulin LifeFactor 5 µg/mL, L-Glutamine LifeFactor 6 mM, Epinephrine LifeFactor 1 µM, Apo-Transferrin 0.5 µg/mL, rh TGF-α LifeFactor 0.5 ng/mL, Extract P LifeFactor 0.4%, and, finally, Hydrocortisone Hemisuccinate LifeFactor 100 ng/mL.

Both cell lines were expanded and passaged at regular periods based on their growth characteristics and in accordance with the protocol of the manufacturers. Incubation was performed at 37 °C, and 5% CO_2_ (Panasonic CO_2_ Incubator, MCO-170AIC, Panasonic Healthcare Co., Ltd., Tokyo, Japan). Discs were placed in 24-well plates in 500 µL GM and incubated for 24 h before seeding the cells. Once confluent, cells were detached using 0.25% Trypsin-EDTA (Gibco, Life Technologies, Inc., Burlington, ON, Canada). Cells were then counted using Millipore Scepter cell counter (Merck Millipore, Watford, UK) and 5 × 10⁴ cells seeded on each disc in a 24-well culture plate (Corning Costar Ultra-Low attachment multi-well plates, Corning Inc., Corning, NY, USA) in 500 µL of complete growth medium. The ratio of the surface area of the sample to medium volume was 3 cm^2^/mL, which is within the ISO standard ratio of 0.5−6 cm^2^/mL, ISO10933 [34].

All cell culture experiments were performed using appropriate controls (growth medium + cells only) with biological and instrumental triplicates (at least 9 discs of each material for each assay) and replicated at least three times. All experiments were executed by (MAO) at Blond McIndoe Laboratories, Stopford Building, Manchester, UK.

### 2.5. Cell Viability

Cellular viability of 100% was attributed to control wells, where cells were cultured with no discs (low control (LC) or positive growth control). Cellular viability was quantified via a colourimetric assay using invitrogen alamarBlue Cell Viability Reagent (Life Technologies Corporation, Thermo Fisher Scientific, Rockford, IL, USA). At each time point, both cell lines were exposed to alamarBlue (1:9 reagent:GM) for 1 h in the incubator (Panasonic CO_2_ Incubator, MCO-170AIC, Panasonic Healthcare Co., Ltd., Tokyo, Japan) at 37° C and 5% CO_2_. Then, 100 µL of supernatant was transferred into a 96-well plate in triplicates for analysis at each time point. Cell viability was measured at four-time points, day 1, 3, 5, and 10 of cell growth. The 96-well plate (Corning Costar Ultra-Low attachment multi-well plates Corning Inc., Corning, NY, USA) was then read with a UVM 340-microplate reader at excitation wavelength of 570 nm and emission wavelength of 600 nm (ASYS, Scientific laboratory supplies). Cell viability was calculated according to the following equation [35]:

$$\mathrm{Cell}\;\mathrm{viability}\;\%=\frac{A570-\left(A600\times \;R_{0}\right)\;\mathrm{for}\;\mathrm{test}\;\mathrm{well}\;}{A570-\left(A600\times \;R_{0}\right)\;\mathrm{positive}\;\mathrm{growth}\;\mathrm{control}\;}\;\times \;100$$

where A570 and A600 are absorbance at 570 and 600 nm, respectively, and R_0_ is the correction factor calculated from (A570/A600) of the positive growth control.

### 2.6. Cytotoxicity

Cytotoxicity of the tested materials was investigated using a CyQUANT LDH Cytotoxicity Assay kit (Thermo Fisher Scientific, IL, USA). Cytotoxicity in both cell lines was measured at 4 time points, day 1, 3, 5, and 10. At each time point, in accordance with the protocol of the manufacturer, 50 µL of lysis buffer (contain membranolytic particles) were added to the specific time point wells (maximum LDH release, high control, HC), 50 µL of sterile-filtered, BioReagent water (SIGMA-ALDRICHᴿ, Life Science, Watford, UK) were added to the low-control wells (spontaneous LDH release), and the plates were incubated at 37 °C in 5% CO_2_ for 45 min. The cytotoxicity was then measured using 50 µL of the supernatant, and 50 µL of LDH cell reaction solution was incubated for 30 min at room temperature in a dark cabinet. The reaction was stopped using 50 µL of the LDH kit stop solution. The 96-well plate (Corning Costar Ultra-Low attachment multi-well plates Corning Inc., Corning, NY, USA) was read with a UVM 340-microplate reader at 490 nm subtracted from 680 nm (ASYS, Scientific laboratory supplies) and cytotoxicity was calculated according to the following equation [36]:

$$\mathrm{Cytotoxicity}\;\%=\frac{\mathrm{Specimen}-\mathrm{treated}\;\mathrm{LDH}\;\mathrm{activity}\;-\;\mathrm{Spontaneous}\;\mathrm{LDH}\;\mathrm{activity}\;}{\mathrm{Maximum}\;\mathrm{LDH}\;\mathrm{activity}\;-\;\mathrm{Spontaneous}\;\mathrm{LDH}\;\mathrm{activity}\;}\;\times \;100$$

where specimen-treated LDH activity is the LDH amount expressed by cells cultured with the discs, maximum LDH activity is the LDH amount expressed by cells treated with lysis buffer, and the spontaneous LDH activity is the LDH amount expressed by cells treated with sterile water.

### 2.7. Statistical Analysis

Data were analysed using statistical software (GraphPad Prism version 9.1.2 (226) (San Diego, CA, USA)) and found to be normally distributed (Shapiro-Wilk’s test). Two-way ANOVA was performed for materials effect, time effect, and their interaction, followed by one-way ANOVA and Tukey’s multiple comparisons, which were performed to compare cell viability and cytotoxicity for different materials at each time point (*p* value = 0.05 for all tests).

## 3. Results

### 3.1. Surface Roughness

*Sa* values (mean—standard deviation (SD)) obtained for materials are presented in (Table 1) below, as well as the maximum and minimum *Sa* values of the three randomly selected points on each disc side.

### 3.2. Surface Morphology Analysis

**Figure 3:**
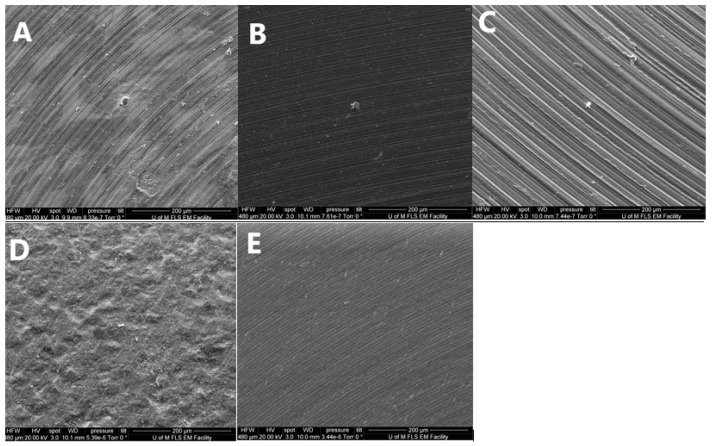
SEM images showing the surface of the investigated materials (**A**): Ti, (**B**): TiN, (**C**): CoCr, (**D**): ZrO_2_, and (**E**): m-PEEK).

The qualitative surface topography analysis (images) of the different materials studied in this experiment are illustrated in Figure 3. Figure 3A shows the machined surface of the Ti material with concentric machining pattern providing different degrees of peaks and troughs. Figure 3B shows a more regular pattern of machining as a TiN layer covering the Ti disc; however, the peaks and troughs of the pattern appear to be less pronounced. Figure 3C shows the CoCr disc surface with a regular pattern and pronounced peaks and troughs. This pattern is not replicated in Figure 3D, where the surface of the ZrO_2_ disc appears to be very granular and irregular, with no defined peaks and troughs. Figure 3E shows the m-PEEK disc surface with the same regular concentric pattern as the other metal discs, although with less pronounced peaks and troughs indicating a smoother surface.

### 3.3. Cell Viability

There was a significant material and time difference in cell proliferation of both HGFB and HGKC cells in contact with all the investigated materials with no significant interaction between different groups.

**Figure 4:**
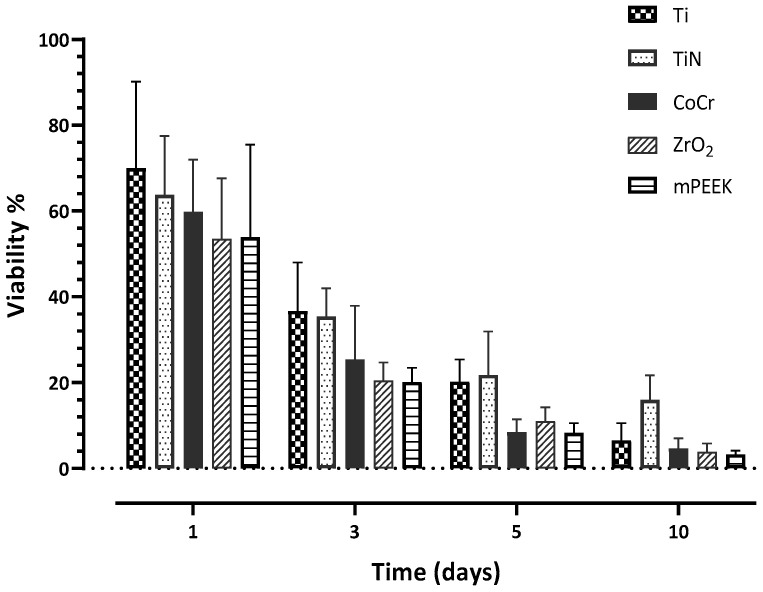
A bar chart illustrating the mean values of cell proliferation (viability) percentage at days 1, 3, 5, and 10 for HGKC. Error bars represent the standard deviation.

HGKC showed the highest cell proliferation when cultured with Ti at day 1 with 69.96%; however, it was not significantly different from the rest of the other investigated materials (Table 2). At day 3, HGKC showed lower proliferation than day 1, with the following order from low to high: m-PEEK < ZrO_2_ < CoCr < Ti < TiN (Figure 4). Notably, HGKC exhibited the lowest proliferation rates with all materials at day 10, with the following order from lowest to highest, which was almost the same order throughout the whole experiment: m-PEEK < ZrO_2_ < CoCr < Ti < TiN (Table 2). In general, TiN showed the highest proliferation rates of HGKC at days 3, 5, and 10 (Table 2).

**Figure 5:**
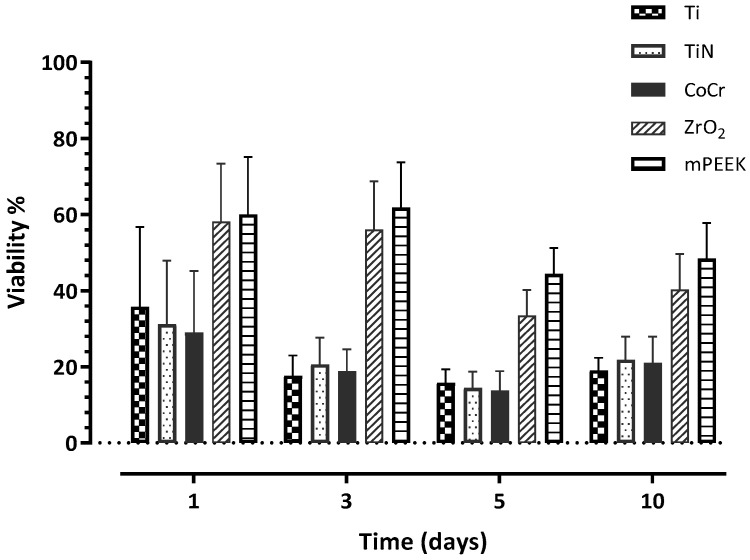
A bar chart illustrating the mean values of cell proliferation (viability) percentage at days 1, 3, 5, and 10 for HGFB. Error bars represent the standard deviation.

HGFB showed the highest proliferation rate when cultured with m-PEEK at day 3, with a proliferation of 61.81%, and it exhibited the highest proliferation with all the other materials at day 1, with the following order from lowest to highest: CoCr < TiN < Ti < ZrO_2_ (Figure 5). HGFB exhibited the lowest proliferation with CoCr, TiN, and Ti at day 3, with proliferation rates of 13.77% < 14.49% < 15.84, respectively. These results were significantly lower than those of ZrO_2_ and m-PEEK at the same time point (Table 3). In general, there was a pattern of high proliferation rates at day 1 for all materials, then the proliferation rates decreased at day 3, especially significantly for Ti, CoCr, and TiN, and reduced further at day 5 for all materials. At day 10, the pattern changed to an increase in the proliferation rate of HGFB in contact with all investigated materials, with m-PEEK showing the highest rate of 48.38% (Figure 5).

Two-way ANOVA for both cell types showed a significant material and time effect (*p* < 0.0001) and nonsignificant interaction.

### 3.4. Cytotoxicity

The results illustrated significant time and material difference as well as a significant interaction between different materials when discs were in contact with HGKC (Table 4). However, there was only significant time difference for HGFB when cultured on different materials. With nonsignificant material difference and no significant interaction between the different investigated groups (Table 4).

**Figure 6:**
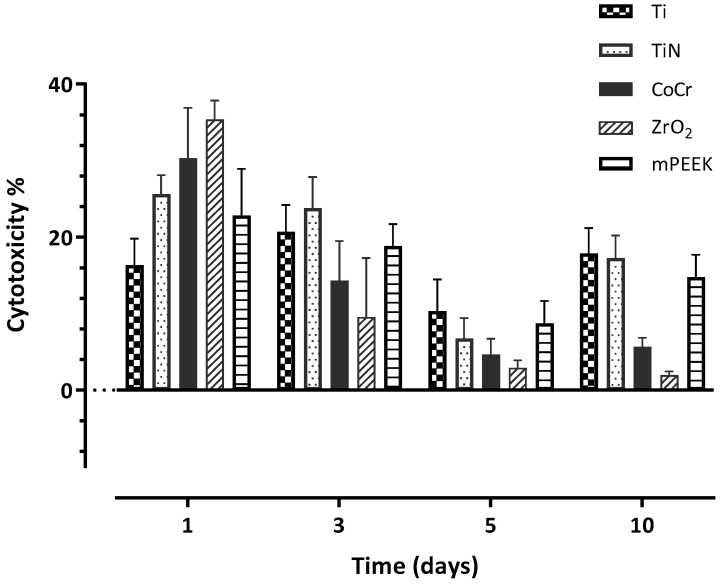
A bar chart illustrating the mean values of cell cytotoxicity percentage at days 1, 3, 5, and 10 for HGKC. Error bars represent the standard deviation.

For HGKC, all material exhibited the highest percentage of cytotoxicity at day 1, apart from Ti when its highest toxicity was at day 3 (Table 4). There was a significant materials and time difference as well as a significant interaction between all the investigated groups throughout the whole experiment, when HGKC was cultured on the discs (Table 4). With time, the cytotoxicity dramatically dropped for all materials at day 3, apart from Ti, which increased by ~4% (Figure 6). Cytotoxicity of the tested materials reached its lowest point at day 5, with the following order from lowest to highest: ZrO_2_ < CoCr < TiN < m-PEEK < Ti with 2.91% < 4.66% < 6.75% < 8.72% < 10.34%, respectively. At day 10, there was an increase in the cytotoxic activity for all materials, except for ZrO_2_, where it further reduced to 1.96% (Table 4).

Two-way ANOVA test showed a significant time and material difference and a significant interaction between different materials (*p* < 0.0001).

**Figure 7:**
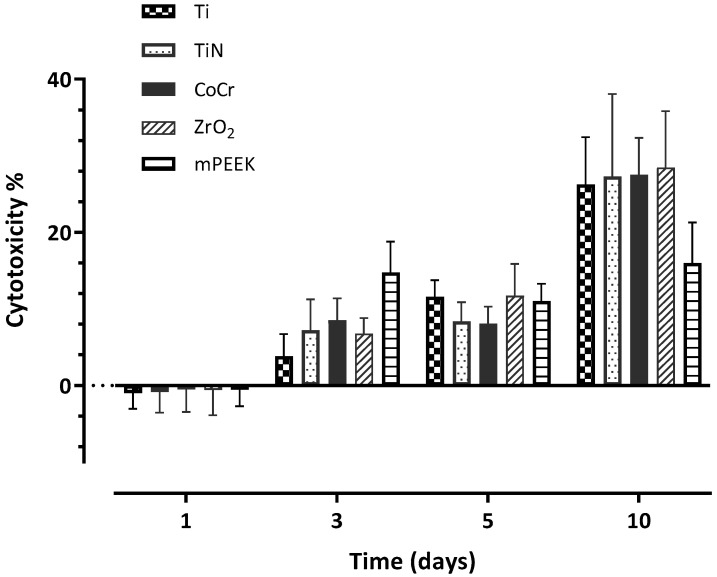
A bar chart illustrating the mean values of cell cytotoxicity percentage at days 1, 3, 5, and 10 for HGFB. Error bars represent the standard deviation.

On the other hand, HGFB showed the lowest cytotoxicity effect for all materials at day 1, in the following order from lowest to highest: Ti < TiN < ZrO_2_ < m-PEEK < CoCr (Table 5). For the following days, the cytotoxic effect of the tested materials on HGFB gradually increased to reach its highest level at day 10 (Figure 7), with the following order from lowest to highest: m-PEEK < Ti < TiN < CoCr < ZrO_2_, with the lowest percentage (15.99%) exhibited by m-PEEK, and the highest (28.46%) exhibited by ZrO_2_ (Table 5).

Two-way ANOVA showed a significant time difference (*p* < 0.0001), nonetheless, there were a nonsignificant material effect and no significant interactions.

**Figure 8:**
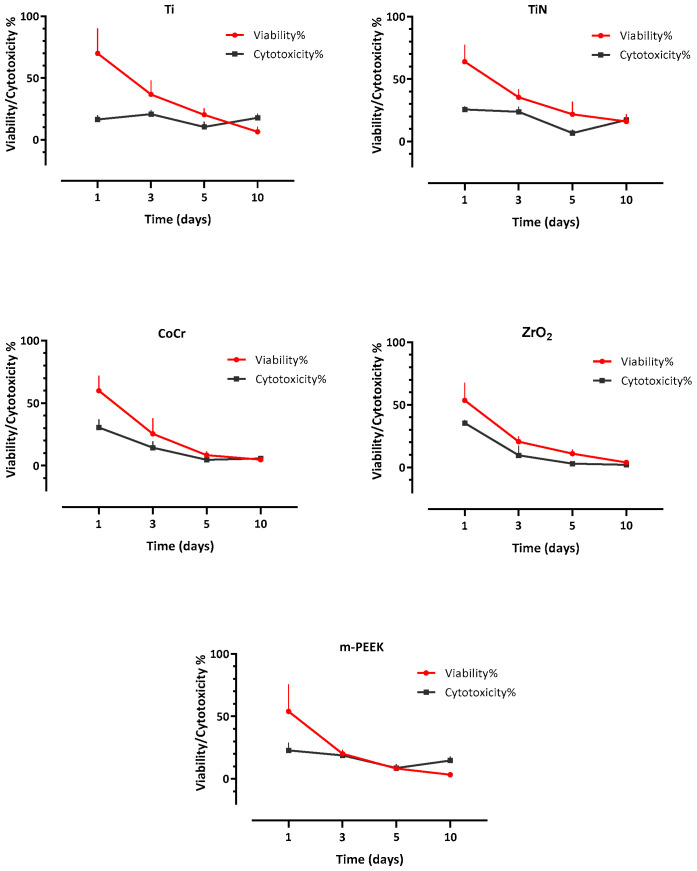
A line plot showing a nonsignificant positive correlation for Ti, TiN, and m-PEEK and a significant positive correlation of CoCr (*p* = 0.004) and ZrO2 (*p* = 0.008) between viability and cytotoxicity over 10 days for HGKC.

**Figure 9:**
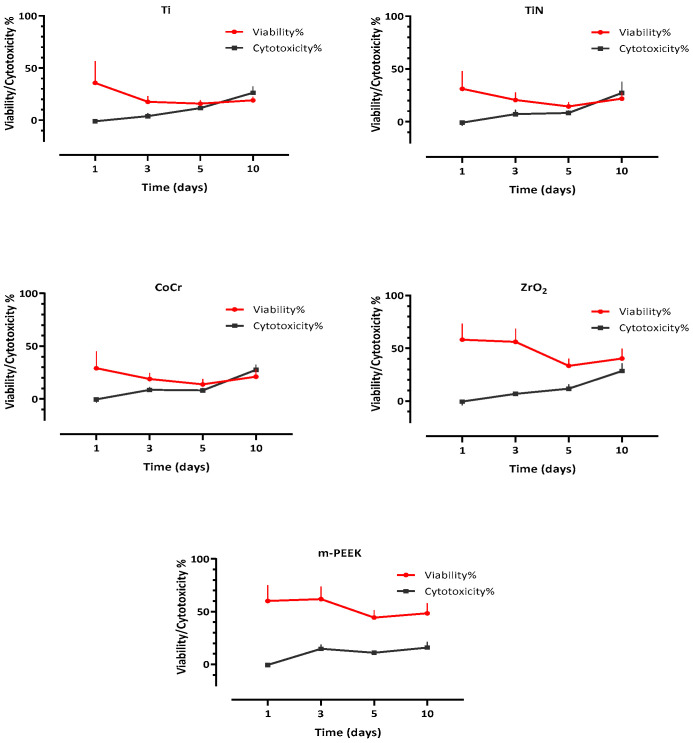
A line plot illustrating a nonsignificant negative correlation between viability and cytotoxicity for all tested materials over 10 days for HGFB.

Both assays were compared for each material over each time point for both cell lines (Figure 8 and Figure 9). In HGKC, a nonsignificant positive correlation for Ti, TiN, and m-PEEK and a significant positive correlation for CoCr (*p* = 0.004) and ZrO_2_ (*p* = 0.008) were found between viability and cytotoxicity. While in HGFB, a nonsignificant negative correlation between viability and cytotoxicity was found for all tested materials.

HGKC cell proliferation was higher than that of HGFB at days 1, 3, and 5 for Ti, TiN, and CoCr. Conversely, cell proliferation of HGFB was higher than that of HGKC throughout the whole experiment for ZrO_2_ and m-PEEK, with m-PEEK exhibiting the highest cell proliferation percentage for HGFB, while TiN exhibited the highest percentage for HGKC at day 10 (Table 2 and Table 3).

These results were generally reversed for cytotoxicity, with HGFB showing an initial very low cytotoxicity that then increased gradually throughout the experiment to reach the end of 20 s for all materials, except m-PEEK (Table 5). On the other hand, HGKC showed initially high cytotoxicity to all materials, which decreased from days 3 to 5 and then increased at day 10 for all materials, except for ZrO_2_, which exhibited the lowest cytotoxicity level at day 10 (Table 4).

## 4. Discussion

In the present study, five implant abutment materials (Ti, TiN, CoCr, ZrO_2_, and m-PEEK) with similar manufacturing techniques, supplier, and degrees of surface roughness were investigated in terms of their influence on HGKC and HGFB proliferation and cytotoxicity.

Previous experiments have investigated the biocompatibility of PEEK for human osteoblast-like cells (MG-63) and concluded that the cell metabolic activity of MG-63 on PEEK was similar to Ti and Zr [37,38]. Ti dental implants are currently regarded as the gold standard in the field of implant dentistry. As with Ti alloy, Zr and PEEK are considered as bioinert materials [38]—when used as a dental implant—and their osseointegration ability is no different. However, the focal point of the current study is the application of m-PEEK as an implant abutment.

It has been reported that the type of the material used, its surface roughness, mechanical properties, wettability, and surface energy all can affect cellular adhesion [39]. In general, PEEK has higher hydrophobicity and lower surface energy than metallic or ceramic materials, due to the presence of fewer polar functional groups on PEEK surfaces, and the results from this study are consistent with this [40]; however, our focus was only on the biological effect of the type and the roughness of the materials used as an implant abutment.

In terms of cell proliferation, the findings of this study showed that HGFB exhibited a better proliferation rate when seeded on m-PEEK and ZrO_2_ discs from day 1 to day 10. Similar results were reported in previous studies, when PEEK was modified with plasma spraying and compared with coated and non-coated Ti, Zr, and other materials and surfaces [28,41]. On the other hand, both materials (m-PEEK and ZrO_2_) exhibited the smallest proliferation percentage at day 10 compared to Ti, TiN, and CoCr, when cultured with HGKC.

Ramenzoni et al. and Paulami et al. [42,43] assessed the consequence of m-PEEK implant abutments on epithelial keratinocyte migration, and they found that all the tested groups were biocompatible, which is the same as the final results we had in this study; however, they both reported that the m-PEEK surface had better cell proliferation and biocompatibility than Ti, TiN-coated, and zirconia, and this was different from our findings which could be due to the saturation of the surface with HGKC and there was no more space on the surface where the cells could proliferate more. The reason for having different results as mentioned by Ramenzoni et al. [42] could be due to “*variations in cell culture times and types of cells and assays used*”, as they checked the viability only at one time point after 24 h [42], and two times in 24, and 48 h [43]. Another reason is that HGKC are very delicate, and their culturing procedure can be challenging. Nonetheless, the cytotoxicity results in this experiment confirms the biocompatibility of m-PEEK (Table 3).

In terms of cytotoxicity, m-PEEK showed the least cytotoxic effect on HGFB (Table 4). However, none of the investigated materials have reached the cytotoxic level set by ISO (30%) throughout the experiment [44]. When cultured with HGKC, m-PEEK exhibited less and slightly better cytotoxic effect than Ti and TiN which is in accordance with results reported in previous studies [43,45].

TiN exhibited comparable to better viability and proliferation rates than Ti and CoCr when cultured with both cell lines. TiN seemed to not alter the original texture of the Ti surface. Previous in vitro studies found that TiN on a Ti alloy showed a higher proliferation rate of fibroblast cells compared with machined uncoated Ti [46,47].

The present study shows that under the same culture conditions for HGFB, m-PEEK and TiN displayed comparable (and at some points better) cell metabolic activity to traditional implant abutment materials. However, when it comes to HGKC proliferation, the results illustrated that TiN exhibited the best proliferation percentage at day 10, while m-PEEK showed the smallest proliferation percentage from day 1 to day 10.

Adhesion is the basic requirement for any living cell(s) to survive on a material. Only then other cellular phenomena, such as cell maturation, migration, proliferation, and differentiation, can occur. This can help with collagen/protein secretion, wound healing, and tissue regeneration, and the same criteria applies to HGBF and HGKC and all cells that come in contact with any implanted device.

The current results showed no significant cytotoxicity when both cells were seeded on all the investigated materials, which suggests that m-PEEK and TiN are materials with similar cytotoxicity to Ti, ZrO_2_, and CoCr. The use of antibiotics is effective in treating bacterial infection; however, they are not routinely prescribed following implant placement for a variety of reasons [46]. Consequently, in the current study, antibiotics were not used during the cell culture process.

The present study indicates that m-PEEK and TiN represent suitable alternative materials to Ti alloy and ZrO_2_ as implant abutments. However, after insertion, many factors can influence the long-term success, such as cell interaction with materials, biofilm formation around abutments, and the stability of materials in an aqueous environment. The current study has only considered HGFB and HGKC; however, other oral cells involved in this process need to be considered in the future.

Fibroblasts were generally found to exhibit significantly higher proliferation rates on comparable surface topographies of ZrO_2_ and Ti, with cells spreading more on polished and machined surfaces than on air-borne-particle abraded surfaces [32], despite the view that R surfaces are believed to provide favourable properties in terms of cellular adhesion for fibroblasts [48]. Studies looking into the same properties with HGKC are lacking, which was one of the reasons why this study was conducted. Surface roughness was not considered as a variable in this experiment, in terms of affecting cellular properties, as it was standardized for all the examined materials.

The long-term success and survival of dental implants may depend not only on the ability of an abutment material to have an appropriate host response and not to cause any biological disturbances but also—in part—on the control of bacterial invasion of the peri-implant and abutment area [49]. The microorganism reservoirs found around dental implants—which are known to contribute to dental implant failure—seem to be interchangeable to the ones identified around natural teeth [49]. Thus, it is equally important to consider the antimicrobial properties of the abutment material, in order to reduce or prevent bacterial adhesion and further accumulation and surface colonization. It has been reported that both surface roughness and wettability (not the focus of this study) have a direct influence on bacterial adhesion and colonization [50,51]. In this study, we have used a similar range of surface roughness for all the investigated materials as a step to eliminate one of the possible confounding factors [52].

To improve the surface response of HGFB and HGKC, special Ti coatings, such as nitride and anatase or oxidation modelling, can also be manufactured around the implant abutment surface and may have additional antibacterial properties [49,53,54]. Thus, future work should focus on understanding the bacterial and tissue interactions to different material surface topography/chemistry, which play an important role in the long-term stability of the peri-implant tissue.

The materials investigated in this work were selected either as they are currently in use as abutments (Ti, ZrO_2_, and CoCr) [55,56,57] or were materials currently under investigation (TiN, and m-PEEK) [42,58] for providing better aesthetic alternatives, but with (potentially) comparable biological influence on the peri-implant hard and soft tissues.

To the best of the authors’ knowledge, this is the first in vitro study to investigate the biocompatibility of five different materials (metal and non-metal) using alamarBlue (proliferation) and LDH (cytotoxicity) assays for a period of 10 days with four-time-point assessment. Thus, we were not able to make any direct comparison with other studies’ results.

The initial cell proliferation percentage for HGKC was comparable between all investigated materials. As the experiment continued, TiN discs illustrated the highest P% among all other tested materials, which was almost comparable throughout the whole experiment.

It has been reported that; TiN surfaces promote surface roughness and hydrophilicity of the Ti surface. Consequently, these surfaces are capable of influencing the gingival cells response. These reports agree with our results, where the biological response of both cell lines used in this study showed the highest P% with HGKC and comparable proliferation rate with Ti and CoCr when cultured with HGFB. TiN also illustrated a low and comparable cytotoxic effect to all the other investigated materials.

In this study, all samples were polished/smooth in order to mimic the trans-gingival surfaces of commercially available abutments. All discs were incubated at 37 °C—after seeding the cells—to simulate intra-oral conditions [59]. Furthermore, the ratio of the surface area of the sample to medium volume was 3 cm^2^/mL, which is within the ISO standard ratio of 0.5−6 cm^2^/mL, for such investigations, ISO 10933, 12 [60].

Another point to highlight is that both HGFB and HGKC cell lines were used in these investigations, as they represent the major cellular component of the soft tissue located within the oral environment [61,62], in direct/indirect contact with the implant abutment, which was considered the most clinically comparable and sensitive method to measure cells viability and low cytotoxicity levels. Finally, in addition to tooth brushing, there is a continuous wash-away of leached-out components from restorative materials by saliva and other consumable fluids in the patient’s mouth; hence, changing the medium could have had a slight washing-out effect.

Preclinica in vitro research is essential for the development of new dental materials and techniques. It can deliver vital data for future tests of therapeutic methodologies in clinical trials [63]. In vitro studies are relatively simple to perform; however, lack of methodologic rigor makes the comparison of results between studies difficult or impossible [64].

The results of the current study should be interpreted in the light of the study’s limitations, including the static in vitro conditions, which lack the dynamics of a living body environment and also the duration of the experiment. In addition, the tested surface roughness of the specimens should be investigated clinically in the oral environment. Whilst both cell lines have been cultured to closely mimic the clinical situation, the current findings should be investigated in vivo.

Another limitation of the current study may be the lack of consideration of inflammation and bacterial endotoxins over the abutment surfaces, as it was done under static conditions. Inflammation and bacterial endotoxins are considered part of the normal oral response, and their presence may indicate limited response due to an acute inflammatory cytokine production. Thus, future cell culture studies should be conducted to comprehensively determine the effect of inflammatory conditions on the relationship between soft tissue cells and biomaterials. In addition, although various reports reinforce the importance of m-PEEK and TiN surfaces on increased osteoblast response, in vitro studies investigating the effect of implant abutment materials on gingival cells, especially HGKCs, are still necessary [42].

## 5. Conclusions

m-PEEK and TiN were observed to have comparable biocompatibility in terms of cell viability, proliferation, and cytotoxicity on both HGKC and HGFB, as compared to uncoated Ti, ZrO_2_, and CoCr.

m-PEEK and TiN surfaces may augment biocompatibility by having a positive impact on the viability and proliferation of HGFB and HGKC as well as a low cytotoxic effect compared to titanium, zirconia, and cobalt-chromium.

Further in vivo evaluations of these materials are necessary to confirm this conclusion.

## Figures and Tables

**Table 1:** *Sa* mean values and SD for Ti, TiN, CoCr, ZrO_2_, and m-PEEK of the three randomly selected pointes on each side of the discs.

Abutment Material	Ti	TiN	CoCr	ZrO_2_	m-PEEK
***Sa*** **value: mean µm (SD)**	0.233 µm (0.018)	0.235 µm (0.012)	0.349 µm (0.014)	0.363 µm (0.013)	0.232 µm (0.021)
**Minimum** ***Sa*** **value**	0.212 µm	0.222 µm	0.340 µm	0.213 µm	0.212 µm
**Maximum** ***Sa*** **value**	0.247 µm	0.247 µm	0.366 µm	0.373 µm	0.255 µm

**Table 2:** The mean and standard deviation values of cell proliferation percentage at days 1, 3, 5, and 10 (HGKC). Values with the same superscript letters represents a non-significant difference (Tukey’s post hoc (*p* > 0.05)) between the investigated materials at each time point.

Materials	Ti	TiN	CoCr	ZrO_2_	m-PEEK
Time					
Day 1	69.96 (20.17) ^A^	63.80 (13.69) ^A^	59.81 (12.13) ^A^	53.50 (14.15) ^A^	53.9 (21.55) ^A^
Day 3	30.69 (11.3) ^A^	35.42 (6.57) ^A^	25.39 (12.53) ^A,B^	20.48 (4.19) ^B^	20.15 (3.29) ^B^
Day 5	20.24 (5.18) ^A^	21.79 (10.12) ^A^	8.43 (2.99) ^B^	10.99 (3.25) ^B^	8.27 (2.28) ^B^
Day 10	6.53 (4.03) ^A^	16.01 (5.70) ^B^	4.69 (2.35) ^A^	3.81 (2.03) ^A^	3.3 (0.87) ^A^

**Table 3:** The mean and standard deviation values of cell proliferation percentage at days 1, 3, 5, and 10 (HGFB). Values with the same superscript letters represents a non-significant difference (Tukey’s post hoc (*p* > 0.05)) between the investigated materials at each time point.

Materials	Ti	TiN	CoCr	ZrO_2_	m-PEEK
Time					
Day 1	35.78 (20.99) ^A,C^	31.16 (16.79) ^A^	29.00 (16.18) ^A^	58.18 (15.2) ^B,C^	60.01 (15.06) ^B^
Day 3	17.65 (5.37) ^A^	20.65 (7.04) ^A^	18.18 (5.7) ^A^	56.09 (12.6) ^B^	61.81 (11.96) ^B^
Day 5	15.84 (3.52) ^A^	14.49 (4.22) ^A^	13.77 (5.12) ^A^	33.40 (6.82) ^C^	44.38 (6.80) ^B^
Day 10	19.09 (3.27) ^A^	21.87 (6.06) ^A^	21.05 (6.92) ^A^	40.27 (9.30) ^B^	48.38 (9.40) ^B^

**Table 4:** The mean and standard deviation values of HGKC cell cytotoxicity percentage at days 1, 3, 5, and 10. Values with the same superscript letters represents a non-significant difference (Tukey’s post hoc (*p* > 0.05)) between the investigated materials at each time point.

Materials	Ti	TiN	CoCr	ZrO_2_	m-PEEK
Time					
Day 1	16.34 (3.45) ^A^	25.62 (2.47) ^B,D^	30.33 (6.57) ^B,C^	35.36 (2.49) ^C^	22.85 (6.06) ^D^
Day 3	20.71 (3.47) ^A,B,D^	23.80 (4.06) ^A,D^	14.33 (5.17) ^B,C,E^	9.56 (7.69) ^C^	18.86 (2.82) ^D,E^
Day 5	10.34 (4.13) ^A,D^	6.75 (2.66) ^A,D^	4.66 (2.04) ^B^	2.91 (1.01) ^C^	8.72 (2.93) ^D^
Day 10	17.86 (3.32) ^A,C^	17.23 (2.97) ^A,C,E^	5.68 (1.15) ^B,E^	1.96 (0.48) ^B^	14.77 (2.93) ^C^

**Table 5:** The mean and standard deviation values of HGFB cell cytotoxicity percentage at days 1, 3, 5, and 10. Means with the same letters are not statistically different (*p* > 0.05).

Materials	Ti	TiN	CoCr	ZrO_2_	m-PEEK
Time					
Day 1	−1.02 (2.03) ^A^	−0.84 (2.70) ^A^	−0.51 (2.92) ^A^	−0.55 (3.33) ^A^	−0.52 (2.16) ^A^
Day 3	3.79 (2.90) ^A^	7.22 (4.03) ^A,C^	8.51 (2.89) ^B,C^	6.80 (2.01) ^A,B^	14.75 (4.01) ^D^
Day 5	11.61 (2.15) ^A^	8.36 (2.51) ^A^	8.07 (2.24) ^A^	11.72 (4.12) ^A^	11.01 (2.27) ^A^
Day 10	26.25 (6.17) ^A^	27.29 (10.77) ^A^	27.51 (4.82) ^A^	28.46 (7.35) ^A^	15.99 (5.27) ^B^

## Data Availability

Data can be requested from the corresponding author.
